# mtDNA analysis confirms the endangered Kashmir musk deer extends its range to Nepal

**DOI:** 10.1038/s41598-019-41167-4

**Published:** 2019-03-20

**Authors:** Paras Bikram Singh, Janak Raj Khatiwada, Pradip Saud, Zhigang Jiang

**Affiliations:** 1 Key Laboratory of Animal Ecology and Conservation Biology, Institute of Zoology, Chinese Academy of Sciences, Beijing, 100101 China; 2 University of Chinese Academy of Science, Beijing, 100049 China; 3 National Trust for Nature Conservation, Khumaltar, Nepal; 4 Chengdu Institute of Biology, Chinese Academy of Sciences, Chengdu, 610041 China; 5 Animal and Range Sciences, New Mexico State University, Las Cruces, NM 88003 USA

## Abstract

Musk deer *Moschus* spp. are endemic to the high mountain forests of central Asia. The taxonomic status of musk deer in the central and western Himalayas is poorly understood. We investigated the phylogenetic relationship of musk deer from the central and western Himalayas based on mitochondrial genomic data of Cytochrome b (380 bps) and D-loop (1000 bps). Our results distinguished two divergent lineages using higher bootstrap support (bs) values from the Maximum likelihood and Bayesian posterior probabilities (bpp). Both the Manang and Kaski lineages from central Nepal are confirmed as Himalayan musk deer *Moschus leucogaster* and represent a species complex widespread throughout the central and eastern Himalayan region. The musk deer Mustang lineage was confirmed as Kashmir musk deer *Moschus cupreus* and has wide distribution in the western Himalayas (from central Nepal to Afghanistan). Our analysis validates that Kashmir musk deer is a genetically distinct species and it clarifies that Himalayan musk deer and Kashmir musk deer are confirmed instead of Alpine musk deer *Moschus chrysogaster* which has been previously described from the southern parts of Himalayas of Nepal, India and Pakistan.

## Introduction

Musk deer *Moschus* spp. are shy, timid, crepuscular and nocturnal and forest dwellers^1–3^. Musk deer are threatened in their montane range of central Asia primarily due to poaching and the illegal trade in musk pod which is used to produce perfumes and traditional medicines. Several musk deer species are in great need of conservation action^4,5^, which are generally inconspicuous to humans. Musk deer establish latrine sites where they defecate to mark territory. These sites can be used to detect the presence and abundance of musk deer in the field and correlate their habitat preference^3,6^. Information collected at latrine sites can be used to prepare and to execute conservation strategies and management actions such as anti-poaching and habitat management. Therefore, for drawing target species-specific conservation strategy, our original aim was to understand latrine use behavior and the habitat ecology of musk deer in the Neshyang valley, Manang, Annapurna Conservation Area (ACA) in the high Himalaya of Nepal. We used trail cameras set on latrine sites and biophysical data collected from musk deer habitat in the Neshyang Valley, Manang (hereafter Manang) to study musk deer activities.

**Figure 1:**
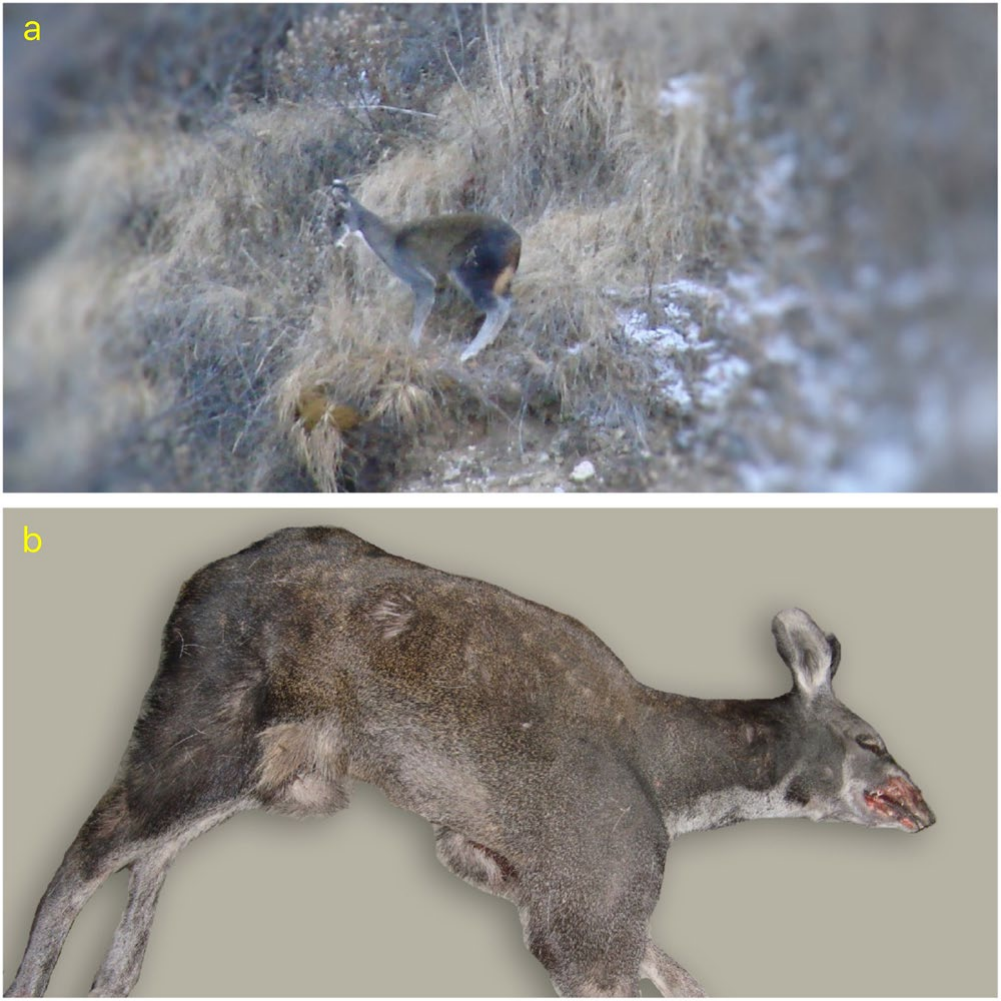
Photographs of unidentified musk deer from Mustang, Nepal. (**a)** Images of a deer in the wild taken 11/14/ 2010; and **(b)** Image of a carcass seized from Marpha, Mustang, Annapurna Conservation Area (Later, these musk deer were confirmed genetically as Kashmir musk deer).

**Figure 2:**
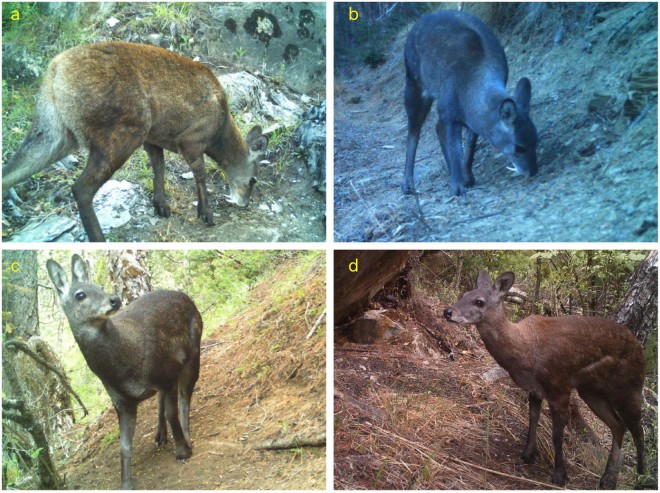
Different pelage color of Himalayan musk deer captured by camera traps in Manang, Annapurna Conservation Area, Nepal during summer and winter. (**a**) Male: 6/24/2017; (**b**) male: 11/23/2016; (**c**) female: 6/15/2017; and (**d**) female: 6/30/2016.

Preliminary analysis using images taken at camera traps indicated that the musk deer in Manang and Mustang were likely different species. We compared the images of musk deer taken by the first author of this paper from the Mustang, Annapurna Conservation Area (hereafter Mustang) in November 2010. Additionally, we also analyzed photographs of the skin and carcasses confiscated from poachers by the ACA office, Mustang. Contrasting evidence of musk deer from Mustang (Fig. 1) and Manang (Fig. 2) indicating different musk deer in the area; thus we determined to further investigate the musk deer in Mustang as well. We also examined the fresh pellets of musk deer from the Kaski area (Fig. 5) molecularly to evaluate the taxonomic relationships of musk deer and their distribution from all habitats in the area in reference to Annapurna Himalayas range. Mustang is located in west, Manang in the east and Kaski in the south of Annapurna Himalayas range.

Species in the genus *Moschus* are cold-adapted large herbivores occurring commonly at higher elevations in central Asia mountains. Because of their cold and high-altitude adaptations to specialized habitat requirements, they are a unique group to make attractive grouping for taxonomic status evaluation. At present seven species of musk deer; Anhui musk deer *M*. *anhuiensis*, forest musk deer *M*. *berezovskii*, Alpine musk deer *M*. *chrysogaster*, black musk deer *M*. *fuscus*, Himalayan musk deer *M*. *leucogaster*, Kashmir musk deer *M*. *cupreus* and Siberian musk deer *M*. *moschiferus* are known to occur in the forests and alpine scrublands of the mountains of Asia^7–10^. Three species; Alpine musk deer, Himalayan musk deer and black musk deer have so far been reported from the Nepal^11–13^. However, these records are primarily based on earlier observations from neighboring Uttarakhand, India and the Tibet Autonomous Region of China (hereafter Tibet) from presence-only surveys or from anecdotal information^8^. Cryptic species like musk deer are extremely difficult to identify using only morphological information. With the advancement of molecular technology in taxonomic research, identifying cryptic taxa has become easier^14^. Six out of the seven species of musk deer have been distinguished in China based on molecular technologies^15^. These technologies are not yet widely applied in south Asia, especially in the Himalayas. Recently, with the introduction of molecular methods in taxonomic studies, several species of fauna have been discovered or re-described from the Nepal Himalaya^16–20^.

From the beginning, our study based on mtDNA indicated that there was discovering a new species of musk deer from Mustang. Our preliminary analysis of the sequence of cytochrome b (Cytb) sequences from the fresh pellets of musk deer collected from Mustang showed different clades compared to the samples at Manang and Kaski. Our excitement increased when the genetic sequence of musk deer from Mustang did not match the genetic sequence of six other known species of musk deer: Anhui musk deer, forest musk deer, alpine musk deer, black musk deer, Himalayan musk deer (hereafter HMD), and Siberian musk deer. However, there was still one species of musk deer that remained to be compared, that was Kashmir musk deer (hereafter KMD). KMD is the only recorded species of musk deer from Kashmir and surrounding regions of India, Pakistan, and Afghanistan and it is also the least known musk deer species^9,21^. Because the KMD has not been intensively studied, there was not a gene sequence for the species in the NCBI gene bank. A scenario was developing indicating that the species found in Mustang could be a new species of the musk deer because of its distinct morphological characteristics and the sizeable geographical distance between Mustang, Nepal and Kashmir, India. Further, recent studies of musk deer from southern parts of Himalaya including Nepal suggested a possibility of new species^22–24^. After we gathered all the preliminary evidences, our team started to follow the deer and setting camera traps on the latrine sites and collecting fresh pellets samples. By using this approach, we hoped to understand genetics of the musk deer found in Mustang and Manang. Here, we describe the presence of *M*. *leucogaster* from Manang and Kaski regions east of Annapurna Himalayas range and for the first time the presence of *M*. *cupreus* in Mustang, Nepal, west of Annapurna Himalayas range based on molecular and camera trap methods.

## Results

### Phylogenetic relationship of musk deer

**Figure 3:**
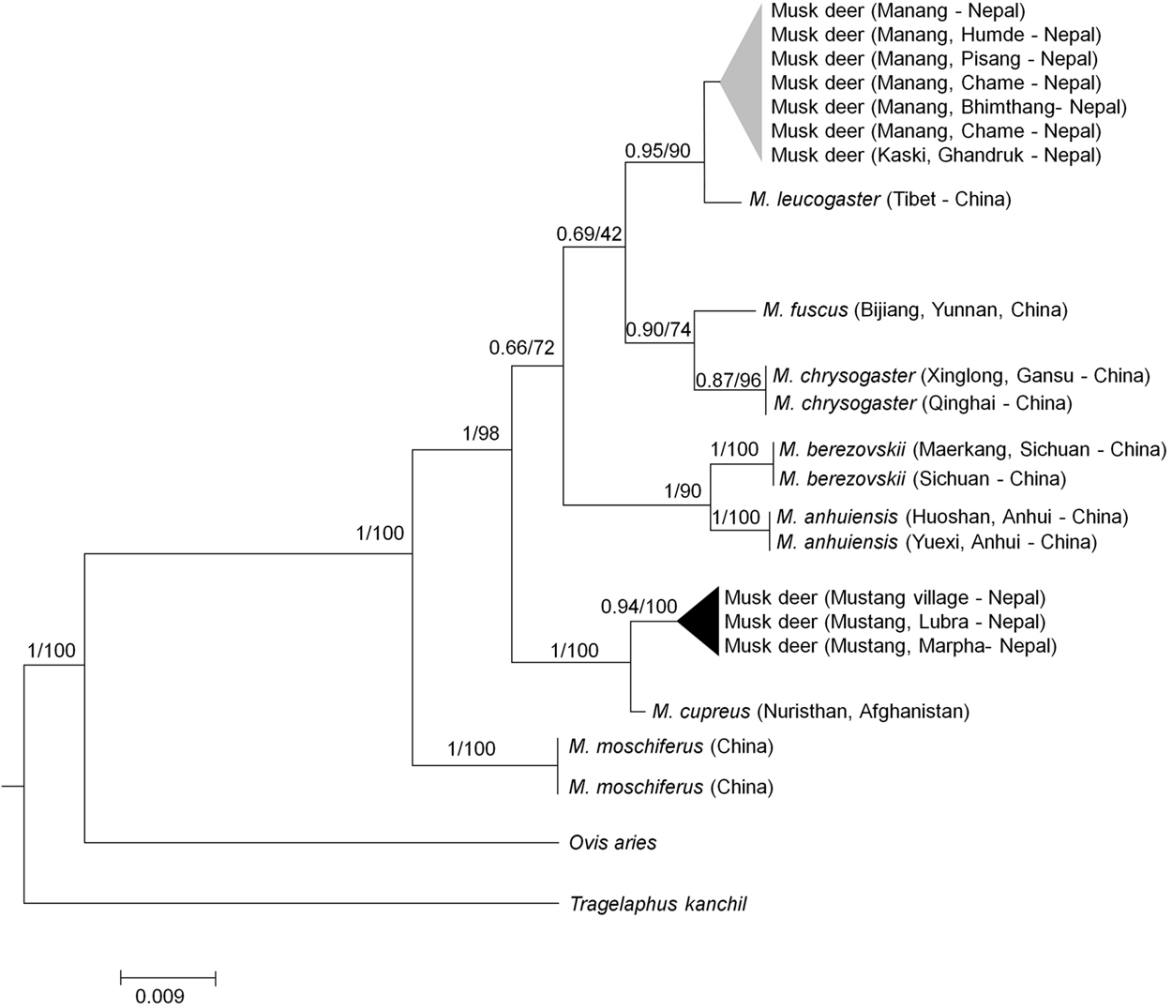
Bayesian inference (BI) tree estimated based on mtDNA Cytb sequences. Values on branches of the tree show the Bayesian posterior probabilities / bootstrap support value for maximum likelihood. Sample names correspond to those given in Table 1. For clarity, branches that representing individuals with the same taxonomic unit were collapsed. Grey triangles refers to *M*. *leucogaster* and Black triangles represents to *M*. *cupreus*.

The aligned dataset of Cytb sequence contained a 380-branch points sequence (bps) including 102 variable sites and 68 parsimony informative sites, and a total of 12 unique haplotypes (4 Cytb haplotypes from Mustang, 6 from Manang and 2 from Ghandruk). These phylogenetic relationships strongly support the genus *Moschus* as a monophyletic clade with higher posterior probability and bootstrap supports (posterior probability, pp = 1 and bootstrap = 99). Molecular data analysis suggests that the population of musk deer in Mustang is genetically similar to the KMD sample collected from Nuristan, Afghanistan and nested together in a BI (Bayesian Inference) tree (Fig. 3). The musk deer populations from the Manang and Kaski districts of the eastern part of the ACA, Nepal were genetically similar to the population of HMD of Tibet and were clustered together in a BI tree (Fig. 3). The uncorrected genetic divergence of the Cytb gene sequences between musk deer population of Mustang, Nepal and Nuristan, Afghanistan was 0.5% (Table 1). The genetic divergences between *M*. *cupreus* and its closest relatives *M*. *leucogaster*, *M*. *chrysogaster*, *M*. *fuscus*, *M*. *moschiferus*, *M*. *anhuiensis* and *M*. *berezovskii* were 8.9%, 7.9%, 8.3%, 8.5%, 9.2% and 10.9% respectively. Whereas, the maximum genetic difference of *M*. *cupreus* was with *M*. *berezovskii* (10.9%) and minimum with *M*. *chrysogaster* (7.9%). Similarly, the uncorrected genetic divergence in the Cytb gene sequences between musk deer population of *M*. *leucogaster* from Manang and Kaski and Tibet was 0.30%. The total alignment of concatenated DNA sequences from mitochondrial markers Cytb and D-loop, and then with the only D-loop gene also yielded identical results in the BI analysis (Supplementary Figs S1 and S2). Based on these phylogenetic results, the musk deer populations of the eastern ACA represent Himalayan musk deer *M*. *leucogaster*, while the populations of the western ACA represent Kashmir musk deer *M*. *cupreus*. All three phylogenetic trees (Figs 3 and S1,S2) showed that *M*. *moschiferus* was at the base of the tree and the remaining six species of musk deer shared a common ancestor. Similarly, analysis from all three phylogenetic trees indicated that *M*. *cupreus* evolved prior to *M*. *leucogaster*, *M*. *chrysogaster*, *M*. *fuscus*, *M*. *berezovskii*. These results show a clustering of musk deer species indicating that *M*. *anhuiensis*. *M*. *leucogaste*r, *M*. *chrysogate*r, *M*. *fuscus* share the same lineage, and *M*. *berezovskii* and *M*. *anhuiensis* are in a sister group.

**Table 1:** Genetic uncorrected p-distance of the mtDNA Cytb sequences of the genus Moschus used in this study.

	Species	1	2	3	4	5	6	7	8	9	10	11
1	*M*. *cupreus (Mustang)*											
2	*M*. *cupreus (Afganistan)*	0.005										
3	*M*. *leucogaster -* Kaski	0.089	0.082									
4	*M*. *leucogaster -* Manang	0.092	0.086	0.003								
5	*M*. *leucogaster* -Tibet	0.092	0.086	0.003	0.003							
6	*M*. *berezovskii*	0.109	0.102	0.076	0.076	0.079						
7	*M*. *anhuiensis*	0.092	0.085	0.060	0.060	0.063	0.014					
8	*M*. *moschiferus*	0.085	0.079	0.072	0.072	0.075	0.085	0.069				
9	*M*. *chrysogaster*	0.079	0.073	0.014	0.014	0.016	0.085	0.069	0.075			
10	*M*. *fuscus*	0.083	0.076	0.016	0.016	0.014	0.082	0.066	0.072	0.008		
11	*O*. *aries*	0.187	0.178	0.157	0.157	0.161	0.190	0.176	0.171	0.161	0.173	
12	*T*. *kanchil*	0.220	0.211	0.202	0.202	0.206	0.223	0.225	0.201	0.216	0.211	0.206

### Musk deer with different pelage colors

At latrine sites, we recorded musk deer in every camera because musk deer visit their latrine sites to defecate and to mark their territories for establishing chemical communications. We recorded a total of 53 photographs (27 of males and 26 of female), including 15 one-minute video in Mustang, 22 images and 7 one-minute videos from Lubra and 31 images and 8 one-minute video from Marpha. Most of the videos and images were captured at dusk, night or dawn. We used 14 images (8 of males and 6 of females and 4 one-minute videos (1 of a male and 3 of females) which were captured in daylight in Mustang in the analysis. A total 781 one-minute videos and 234 photographs were collected from trail cameras set in Manang to study behavior at latrine sites. Of these, 20 images (12 of males and 8 of females) and two one-minute videos (1 of a male and 1 of a female) were used to identify pelage color. All of the images and videos from Manang and Mustang were taken between 8:00 hrs. to 12:00 hrs. in the morning and 15:00 hrs. to 17:30 hrs. in the evening. These photographs had similar patterns of light and were free of tree shadows. The pelage color of KMD and HMD were noted from selected photographs (Table 2).

**Table 2:** Comparison of body characteristics of Kashmir musk deer with other six musk deer.

Characteristics	*M. cupreus*	*M. leucogaster*	*M. berezovskii*	*M. chrysogaster*	*M. muschiferus*	*M. fuscus*	*M. anhuiensis*
Front Head (below eye)	Grey	Grey black	Grey	Grey	Grey	Black	Grey
Crown (Fore head hair)	Grey and coppery red	Grey black	Dark grey	Pale brown	Grey brown	Black	Grey
Ear (outside)	Dark brown	Grey brown	Black	Pale brown	Dark brown	Black	Dark
Ear (inside	White and grey black	Grey	Whit	Grey	White	White	White
Ear base	White (frosted look)	Grey	Orange	Pale brown	Grey brown	Black	Grey
Ear tip (outside)	Coppery red	Grey	Black	Yellow	Pale brown	Black	Dark
Throat	White	No strip (Grey)	Three wide strips	White strip	White strip (a pair)	No strip	Strip
Neck (underside)	White	No strip (Grey)	Three wide strips	White strip	White strip	No strip	Strip
Chin	Grey	Grey	White	White	White	Black	Light grey
Nape	Coppery red	Grey brown	Grey brown	Yellowish brown	Dark brown	Black	Grey brown
Thigh	Coppery red	Dark	Dark grey	Paler than black	Black brown	Black	Grey brown
Lower limbs	White	Dark	Grey	Paler than body	Grey	Black	Light grey
Upper limbs	Coppery red and grey	Dark	Grey brown	Greyish yellow	Grey	Black	Grey brown
Dorsal (trunk)	Coppery red (Vaguely white spot)	Black brown	Grey brown	Yellowish brown	Black brown (whitish spot)	Black	Grey brown (Pale spot)
Ventral (trunk)	Coppery red	Grey black	Grey brown	Yellowish brown and grey	Grey brown	Black	Grey brown
Rump	Dark grey	Dark	Nearly black	Paler than black	Black brown	Ocher-y tones	Grey brown

Source^22–24^.

**Figure 4:**
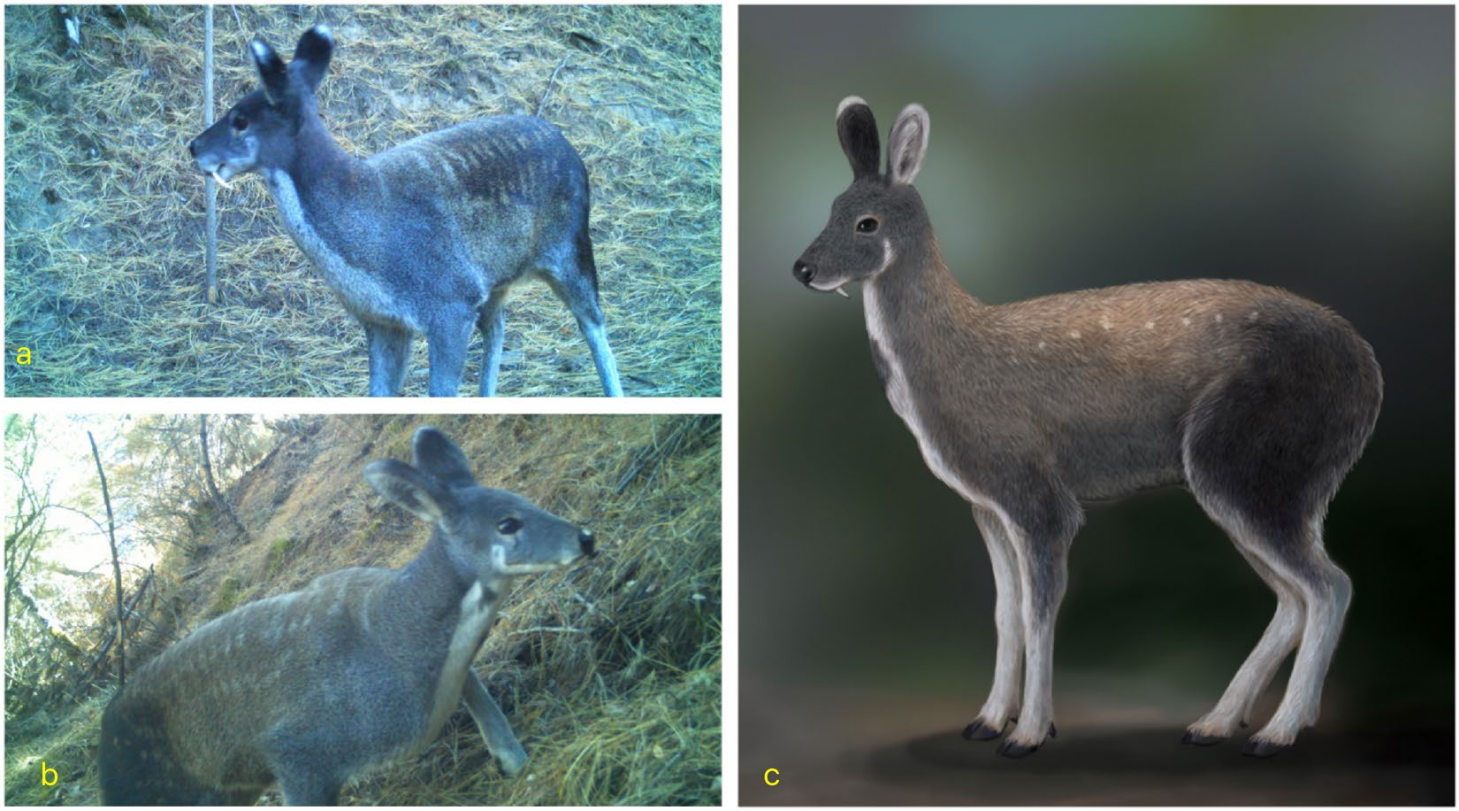
Kashmir musk deer captured by camera traps in the Mustang, Annapurna Conservation Area, Nepal. (**a**) Male 10/27/2017; **(b)** female 11/16/2017; and **(c)** Composite of Kashmir musk deer based on images from Mustang.

These two species had very different pelage coloration which were highly variable in pelage color compared to each other. The KMD had a distinctive pelage color compared to all other musk deer species (Table 2). It has a white color patch running from chin to chest; a black spot on the throat; light brown nape; a light copper colored dorsal surface of the trunk and vaguely spotted; lower parts of the limbs are white; front inner edge of the thigh and rump are predominantly black; and the ears outside are grey-black tipped with white. In contrast to KMD, HMD has a black brown coat and dark legs. It lacks white patches on the neck, and only the juveniles were vaguely spotted (Table 2). Pelage colors varied between individuals of HMD (Figs 2 and S3.). The images of musk deer from summer (May-July 2017) depicted brown color and black color in winter (October-November 2016). Moreover, some images showed light white patches on the neck (Supplementary Fig S3.). KMD from Mustang had a light copper color on the dorsal surface and white patches on the neck. Dorsal surfaces of individuals from both species were vaguely spotted and the lower parts of the limps were white (Fig. 4).

## Discussion

This study provided evidence of the presence of HMD and KMD from Nepal based on molecular and camera trap methods. Previous studies related to habitat and ecology of musk deer from the southern part of the high Himalayas in Nepal, India, and Pakistan^8,11,12,25–29^ have misidentified the Kashmir musk deer as alpine musk deer. All of the studies previously conducted in ACA have also considered the species of study to be alpine musk deer^8,30,31^. The potential misidentification of musk deer is directly partially due to its behavior. Musk deer are extremely shy and nocturnal and therefore, are hard to detect directly^2^. However, observing musk deer in daylight is difficult because they hide in shrub understory during the day^32^. When musk deer are encountered in the forest, they are generally only seen for a few seconds, or only the sound of leaps or escape through bushes can be heard. Green *et al*. (1985) sighted musk deer 74 times in three years of his study in Kedarnath Wildlife Sanctuary, India, and all observations were less than a minute. Sathyakumar (1994) and Vinod and Sathyakumar (1999) sighted musk deer 92 and 65 times respectively during their three year study of musk deer in India.

Taxonomic classification of *Moschus* has remained controversial throughout the range of musk deer and until now unresolved for four decades because these highly cryptic species are extremely difficult to identify reliably using only morphological characters^33–36^. Even under the best conditions, alpine musk deer, Siberian musk deer, and forest musk deer cannot be distinguished by morphology, biogeography or ecological criteria^37^. Three species; Anhui musk deer, forest musk deer and black musk deer can easily be confused with each other because of their similar morphology and pelage color. Often, these species are considered sub-species either of alpine musk deer, Siberian musk deer or forest musk deer^35,38,39^. Recent genetic studies have confirmed that these species are different species of musk deer^15,34^. Guo *et al*., (2018) confirmed genetically that Himalayan musk deer was misidentified as alpine musk deer in Tibet. Before genetic study, Anhui musk deer was considered as sub-species of Siberian musk deer^37^. Pelage color may or may not vary among different species. Alpine musk deer, HMD and KMD are also looked very similar. Pelage may even be different within individuals of any species of musk deer throughout different seasons^23,24^. Investigation of the images captured during this study also showed that different pelage colors in different individuals of HMD and KMD. The reason that earlier researchers misidentified musk deer species in Nepal including in ACA was that they were able to rely on observations of pelage color and morphology.

Although, molecular studies on musk deer have been conducted from their distribution ranges in China^15,22–24,38,40^ this is the first molecular study of musk deer from the southern parts of Himalayas. This study established KMD as a new species recorded in Nepal from Mustang as well as genetically validating it as a distinct species. Liu and Groves, 2014 provided the requisite of DNA verification to establish KMD as a species. This study has also inferred the existence of HMD only east of Mustang from Kaski and Manang along the southern part of the Himalayas.

**Figure 5:**
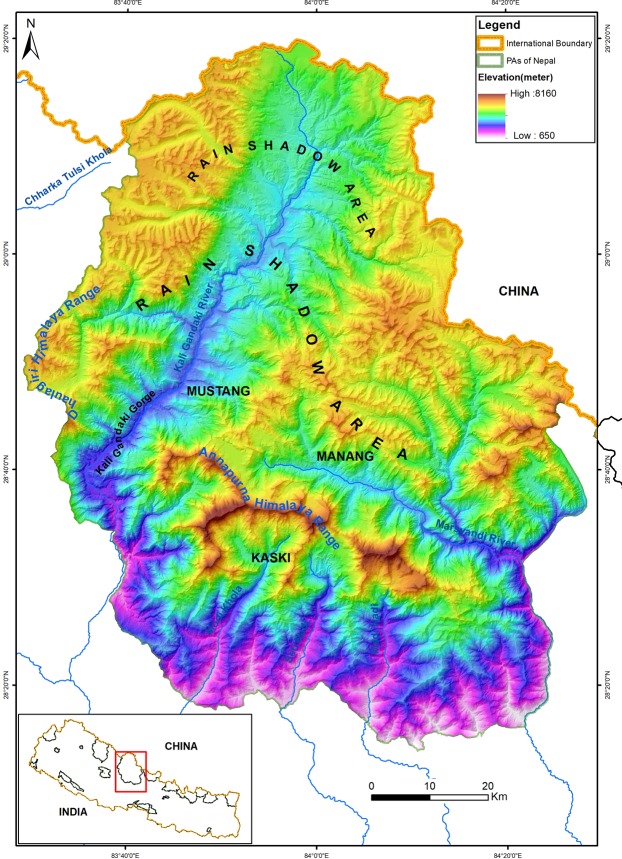
Study area: Annapurna and Dhaulagiri Himalayas, Kaligandaki river and gorge, rain shadow areas, and sample collections sites in Annapurna Conservation Area (Kaski, Manang and Mustang). The map was plotted using ArcGIS 10.3(ESRI, Redlands,CA,USA, http://www.esri.com/).

The Mustang area within the Kaligandaki river gorge and the Annapurna -Dhaulagiri Himalayan range within ACA have likely isolated population of musk deer species in the southern slopes of the Himalaya (Fig. 5). The Mustang valley lies on the southern margin of the Tibetan Plateau and on the leeward side of the high Himalayas. The Kaligandaki river crosses the central Himalayan belt between the Annapurna Himalayas (6993–8091 m asl) and the Dhaulagiri Himalayas (6919–8182 m asl) down to 5,751 m dissecting the eastern and western Himalayas and forming the deepest Kaligandaki gorge in the world^41,42^. To the north, dry habitat in the rain shadow area on the north side of the valley may have prevented the dispersion of musk deer in Tibet. Similarly, Annapurna I (8091 m asl) and its associated Himalayas (Annapurna II, Annapurna III and Annapura IV), glacier lake (Tilicho Lake, 2.98 km^2^), Tilicho Peak (7,134 m asl), and Nilgiri mountain (7,061 m asl) have segregated the forest habitat of Mustang from Manang and Kaski. Geographical barriers; such as mountains, rivers, and gorges obstruct gene flow and lead to genetic differentiation of naturally isolated populations^43–45^. The southern Himalaya, west of Mustang receive active winter precipitation from the Mediterranean while summer precipitation dominates east of Mustang^46,47^. This different precipitation pattern may have resulted in adaptation to different vegetation and climate conditions resulting in the evolution of two distinct species; KMD to the west and HMD to the east of the Annapurna Himalayas and Kaligandaki gorge.

Before this study, KMD had only been described from Kashmir and the associated region of India, Pakistan and eastern Afghanistan^48^. The western distribution of KMD is up to Nuristan, northeast Afghanistan^9^ while the eastern distribution was assumed to be the Kashmir region in India. Our study confirms that the eastern limit of this KMD is Mustang, ACA, Nepal. It suggests that the species of musk deer found in between the two extreme limits in the northern Himalayas of India, Pakistan, and western Nepal must be KMD, though previously they have been identified as alpine musk deer. Our study also showed that Manang and Kaski, Nepal, which lie east of Mustang form the western limit of HMD. A recent genetic study by Guo *et al*., (2018) revealed that HMD from Lazi county, Tibet is close to the range of musk deer from eastern Nepal. In the 2016 the IUCN Red List^49^ has mentioned that the range of alpine musk deer borders eastern Nepal and Bhutan extending from central China, and range of HMD along the entire southern part of Himalayas. However, two species with overlapping niches generally do not coexist and one is displaced by the other^50^. Our study confirmed the presence of KMD westward from Kaski and Manang and HMD eastward from Mustang, with the Annapurna Himalayas and Kaligandaki river gorge as the border between these two species of musk deer. Our study provides the evidence to redefine the existing distributional range of musk deer in the southern part of the greater Himalayas. Based on our molecular analysis and the geographical location of KMD and HMD, we have concluded that alpine musk deer do not exist in Himalayas of Nepal or in northwest India and Pakistan; it is doubtful that they inhabit in Bhutan. Further molecular study of musk deer from Bhutan is warranted to verify the existence of alpine musk deer. It is now apparent that alpine musk deer is found only in central China and HMD is endemic to the eastern Himalaya and KMD in the western Himalaya. Of the seven musk deer species, only Siberian musk deer is not endangered, and all musk deer are endemic to Asia. We believe that a review of the geographical distribution of musk deer from the great Himalayas and China is needed with simultaneously review of their IUCN red list status. Moreover, our research at the beginning was misleading us to define the musk deer in Mustang as a new species of musk deer based on morphological information. This study has finally pointed out significance of molecular analysis in species identification. It is also a useful reminder that only using morphological observation methods are unreliable for the species identification. Therefore, we further recommend molecular based identification of vertebrates in the Himalayas and other regions of the world.

## Materials and Methods

### Study area

The Annapurna Conservation Area (ACA) is located in the western region of Nepal (28°13′ 48″ to 29°19′ 48″ N and 83°28′ 48″ to 84°26′ 24″ E). The ACA covers 7,629 km^2^ encompassing the five districts, Myagdi, Lamjung, Kaski, Manang, and Mustang, and is the largest protected area in Nepal. It has a wide range of habitats from subtropical forest to alpine tundra^51,52^. The area is situated between the Palearctic and Oriental realms and covers the western and eastern Himalayan eco-regions. The ACA covers the Annapurna Himalayas from the south and north, and stretches north to the Tibet Autonomous Region of China (hereafter Tibet). It extends to Manaslu Himalaya (8,163 m asl) in the east and Dhaulagiri Himalaya (8,167 m) in the west. The ACA contains diverse habitats and climates^53^ and harbors 105 species of mammals, 488 species of birds, 20 species of fish, 23 species of amphibians, 40 species of reptiles, and 347 species of butterfly^54^. Three study sites; Manang, Mustang, and Kaski, were selected using Himalaya range and river gorges as a geographical barriers between the populations of musk deer located in these three sections of ACA (Fig. 5).

### Collection of pellets

**Figure 6:**
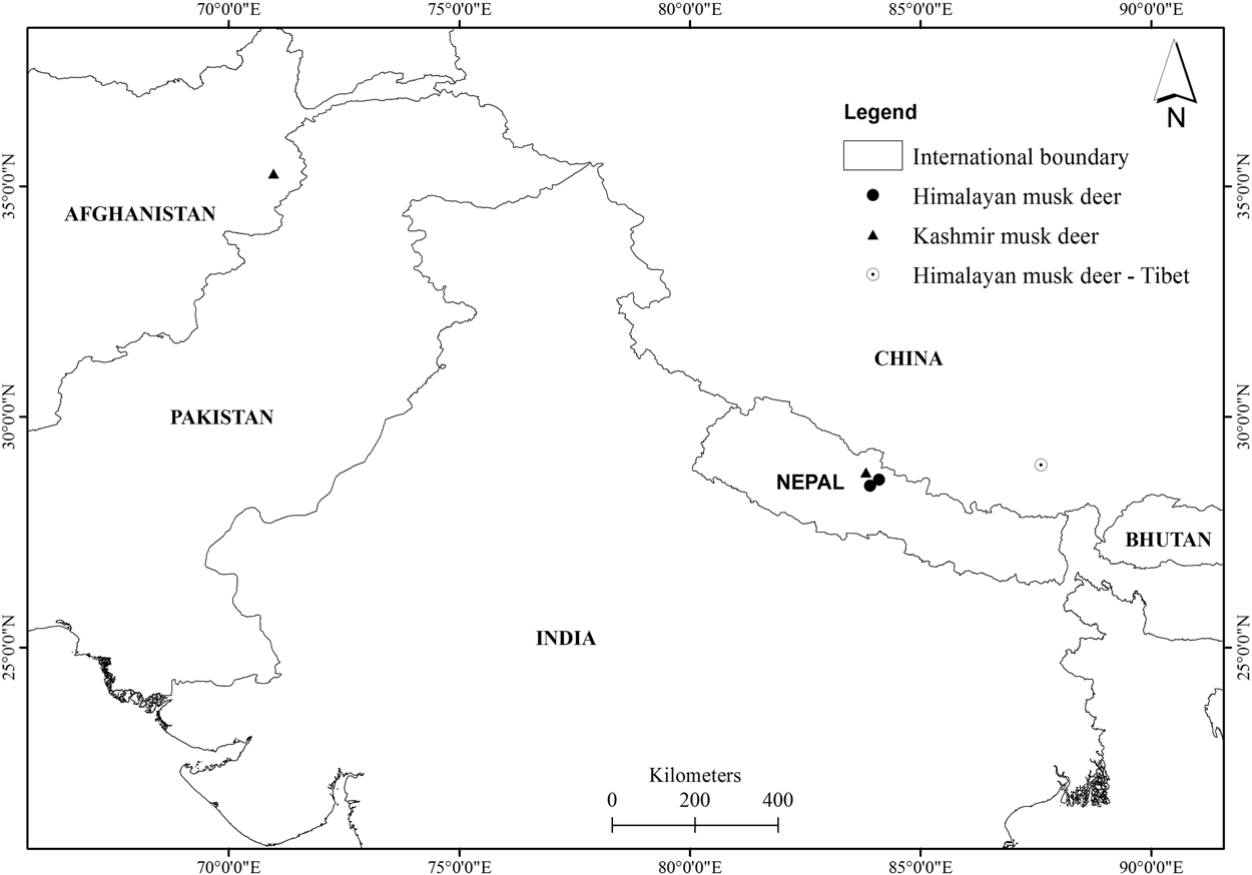
Locations of sampling sites in western and central Himalaya showing confirmed locations of the presence of Kashmir musk deer and Himalayan musk deer. The pointed circle shows the location of the Himalayan musk deer genetically confirmed in Tibet (Guo *et al*., 2018). The map was plotted using ArcGIS 10.3 (ESRI, Redlands, CA, USA, http://www.esri.com/).

To obtain materials for genetic analysis, we collected pellets samples from the Manang, Mustang, and Kaski regions of the ACA during November-December 2016. We collected a total of 300 pellet samples from the study sites. We preserved pellet samples in 95% ethanol and transported them to the molecular laboratory of the National Trust for Nature Conservation in Sahurah, Chitwan Nepal for further molecular analysis, where pellets samples were stored in refrigerator at 4 °C. Additionally, 11 skin samples (ten from Manang and one from Mustang) were obtained from the dry skins of musk deer that died from predation and other natural causes collected by the ACA staffs during forest patrolling in different years from 2009 to 2015. We also used a dry skin sample of Kashmir musk deer that was made available to us from Nuristan, north-east Afghanistan which was collected in July 2009 by the survey team led by Dr. Stephen Ostrowski (Fig. 6).

### Camera trapping

After collecting pellets from latrine sites, we installed cameras (Cuddeback; Green Bay, USA and Bushnell; Overland, USA) at latrine sites of musk deer in Marpha (28.772888°N and 83.665625°E) and Lubra (28.786145°N and 83.817803°E) of the Mustang Valley. The cameras were operational between October and December 2017. We used them to compare color pelage of musk deer. The sites were 19.5 km apart. Latrine sites provided the most reliable pellet collection sites because musk deer visit latrine sites frequently to mark territory by defecating^1,3^. Five camera stations were selected at each site so that the distance between the stations would be a least 0.5 km. Cameras were positioned 35 cm above ground and 2.5 m away from the latrine sites so that both the flanks and head of each animal could be captured in the camera traps. We set fifteen cameras to take five images per trigger, and five cameras were to take a one-minute video. We also installed 18 cameras in Dhikurpokhari (28.597980°N and 84.181286°E), Pisang (28.606472°N and 84.154593°), Ghyaru (28.636689°N and 84.144726°E), Humde (28.633125°N and 84.091719°E), Manang (28.651769°N and 84.021669°E) and Khangsar (28.664650°N and 83.979940°E) of Manang during May-August 2016/2017 and October to December 2016. These sites were at least 3.5 km away from each other.

### DNA extraction, PCR amplification and sequencing

A total of 62 samples (56 pellets sample and 6 skin samples) were selected randomly out of 311 samples stored in the molecular laboratory of National Trust for Nature Conservation to ensure that they represented a large area in the Manang, Mustang and Ghandruk regions of ACA. DNA was extracted from the 60 samples (17 from Mustang, 15 from Kaski and 28 from Manang). In order to maximize DNA concentration in the extraction, we placed ten pellets from each sample in a Petri disc and left them for 5 minutes at room temperature to evaporate ethanol. The outer layer of each pellet was peeled into smaller pieces using sterile scissors as samples for analyses. Then, DNA extractions from the samples were analyzed as per the manufacturer’s instructions for both pellet and tissue (skin) samples using a DNeasy Stool and Tissue Kit (QIAGEN).

Polymerase Chain Reaction (PCR) was used to amplify the mitochondrial genes Cytochrome b (Cytb) and D-loop from all 60 samples. The primers and PCR conditions were used as described by Pan, *et al*.^15^, Zhang and Jiang^55^. PCR products were visualized in 1.5% agarose gel. A total of 44 positive samples (14 from Mustang, 11 from Kaski and 19 from Manang) were sequenced following bi-directional sequencing from ABI 3100 automated sequencer.

### Sequence analysis

Nucleotide sequences of the Cytb gene and D-loop from six of the seven *Moschus* species were downloaded from the NCBI GenBank database and also data source made available by Pan, *et al*.^15^ (Table 3) where sequence of KMD was not available. Nucleotide sequences were assembled by SeqMan and then visually checked to determine the accuracy of the variables site identified by the program. All the sequences were then aligned with ClustalW in BIOEDIT Version 7.1.9^56^ using the default settings. Newly determined sequences were deposited in GenBank under accession numbers (MK363267–MK363317). We developed a phylogenetic tree analysis of all sequences using Bayesian Inference (BI) and Maximum Likelihood (ML). We employed Bayesian Inference analyses to reconstruct the phylogenetic relationships among the taxa based on haplotypes of the Cytb gene sequence datasets. We used The GTR + I + G model of best-fit nucleotide substitution under the Bayesian Information Criterion (BIC) by using the program jModeltest 2.1.4^57^. We carried out Bayesian analyses in the MrBayes 3.1.2^58^. We initiated two dependent runs each with four simultaneous Markov Chain Monte Carlo (MCMC) chains for 30 million generations and sampled every 1000 generations. We examined the convergence of chains and a burn-in period of all runs by plots of log-likelihood scores and low standard deviation of split frequencies. We discarded the first 25% generations as burn-in, and used the remaining trees to create a 50% majority-rule consensus tree and to estimate Bayesian Posterior Probabilities (BPP). We performed Maximum Likelihood analyses in RaxML^59^ and bootstrap supports (bs) for nodes of the resulting ML tree were evaluated by analyzing 1000 bootstrap replicates. We constructed ML tree using Figtree^60^.

**Table 3:** GeneBank accession number of specimens used in phylogenetic analysis.

Species	Locality	GeneBank Accession No
*M*. *leucogaster*	Manang District, Nepal	MK363267
*M*. *leucogaster*	Manang District, Nepal	MK363268
*M*. *leucogaster*	Manang District, Nepal	MK363269
*M*. *leucogaster*	Manang District, Nepal	MK363272
*M*. *leucogaster*	Manang District, Nepal	MK363273
*M*. *leucogaster*	Manang District, Nepal	MK363274
*M*. *leucogaster*	Manang District, Nepal	MK363275
*M*. *leucogaster*	Humde, Manang District, Nepal	MK363276
*M*. *leucogaster*	Humde, Manang District, Nepal	MK363277
*M*. *leucogaster*	Humde, Manang District, Nepal	MK363278
*M*. *leucogaster*	Chame, Manang District, Nepal	MK363279
*M*. *leucogaster*	Chame, Manang District, Nepal	MK363280
*M*. *leucogaster*	Chame, Manang District, Nepal	MK363281
*M*. *leucogaster*	Chame, Manang District, Nepal	MK363282
*M*. *leucogaster*	Pisang, Manang District, Nepal	MK363283
*M*. *leucogaster*	Pisang, Manang District, Nepal	MK363284
*M*. *leucogaster*	Pisang, Manang District, Nepal	MK363285
*M*. *leucogaster*	Bhimtang, Manang District, Nepal	MK363286
*M*. *leucogaster*	Bhimtang, Manang District, Nepal	MK363287
*M*. *cupreus*	Marpha, Mustang district, Nepal	MK363288
*M*. *cupreus*	Marpha, Mustang district, Nepal	MK363289
*M*. *cupreus*	Marpha, Mustang district, Nepal	MK363290
*M*. *cupreus*	Marpha, Mustang district, Nepal	MK363291
*M*. *cupreus*	Lubra, Mustang District, Nepal	MK363292
*M*. *leucogaster*	Ghandruk, Kaski District, Nepal	MK363270
*M*. *leucogaster*	Ghandruk, Kaski District, Nepal	MK363271
*M*. *anhuiensis*	Huoshan, Anhui Province, China	NC020017
*M*. *anhuiensis*	Yuexi, Anhui Province, China	KP684124
*M*. *berezovskii*	Maerkang, Sichuan Province, China	EU043465.
*M*. *berezovskii*	China	NC012694
*M*. *moschiferus*	China	JN632662
*M*. *moschiferus*	China	NC013753
*M*. *chrysogaster*	Gansu Province, China	KC425457
*M*. *chrysogaster*	Qinghai Lake, Qinghai Province, China	KP684123
*M*. *fuscus*	Bijiang, YunnanProvince, China	AF026888
*M*. *leucogaster*	Tibet, China	AF026889
*Ovis aries*	*NA*	NC001941
*Tragulus kanchil*	*NA*	JN632709

## Supplementary information

List of the supplementary figures

## Data Availability

Data will be available on NCBI GeneBank from March 31, 2019.
