# Supplementary material for: mtDNA analysis confirms the endangered Kashmir musk deer extends its range to Nepal
Source: Sci Rep. 2019 Mar 20;9:4895. doi: 10.1038/s41598-019-41167-4 (PMC6426878; doi:10.1038/s41598-019-41167-4)
Supplement: Supplementary file 1 — List of the supplementary figures [file 41598_2019_41167_MOESM1_ESM.pdf]

mtDNA analysis confirms the endangered Kashmir musk deer extends its range to Nepal

Paras Bikram Singh[1,2,3], Janak Raj Khatiwada[4], Pradip Saud[5], Zhigang Jiang[1,2*]

[1] Key Laboratory of Animal Ecology and Conservation Biology, Institute of Zoology, Chinese Academy of Sciences, Beijing 100101, China, [2] University of Chinese Academy of Science, Beijing 100049, China, [3] National Trust for Nature Conservation, Khumaltar, Nepal, [4] Chengdu Institute of Biology, Chinese Academy of Sciences, Chengdu 610041, China, [5] Animal and Range Sciences, New Mexico State University, Las Cruces, NM, 88003, USA

* Corresponding author. *E-mail address*: jiangzg@ioz.ac.cn

List of the supplementary figures

Supplementary Fig. S1. Bayesian inference (BI) tree estimated based on concatenated DNA sequence from Cytb and D-loop. Values on branches of the tree show the Bayesian posterior probabilities / bootstrap support value for Maximum likelihood. Sample names correspond to those given in Table 1. For clarity, branches that representing individuals with the same taxonomic unit were collapsed. Grey triangle refers to *M. leucogaster* and black triangle represents to *M.cupreus*.

Supplementary Fig. S2. Bayesian inference (BI) tree estimated based on D-loop sequences. Values on branches of the tree show the Bayesian posterior probabilities / bootstrap support value for Maximum likelihood. Sample names correspond to those given in Table 1. For clarity, branches that representing individuals with the same taxonomic unit were collapsed. Grey triangle refers to *M. leucogaster* and Black triangle represents to *M.cupreus*.

Supplementary Figs S3. Different pelage color of Himalayan musk deer photographed in Manang, Annapurna Conservation Area, Nepal during summer and winter. (a) Adult male **(b)** Adult male **(c)** Adult female **(d)** Adult male **(e)** Juvenile male **(f)** Juvenile female

Supplementary Figs S4. New distribution of species of musk deer

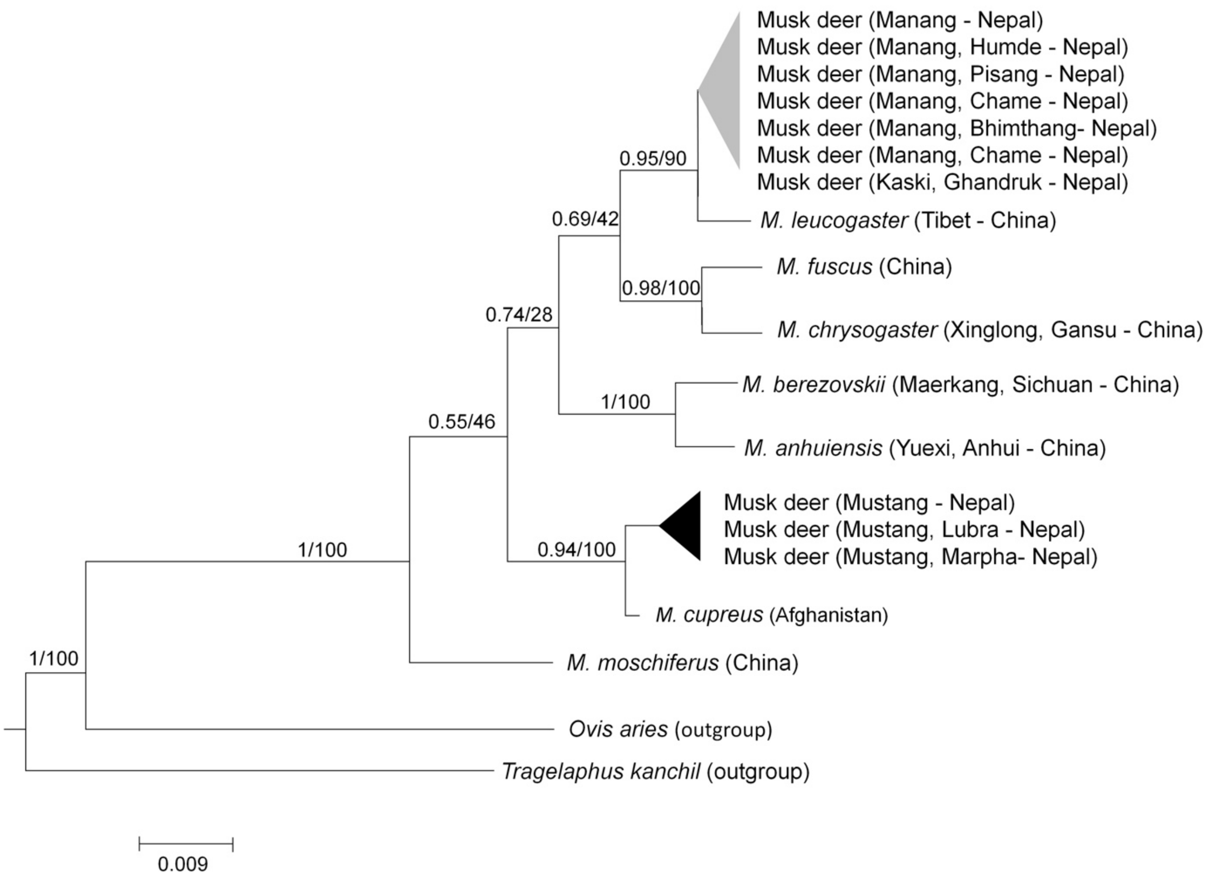

Supplementary Fig. S1. Bayesian inference (BI) tree estimated based on concatenated DNA sequence from Cytb and D-loop sequences. Values on branches of the tree show the Bayesian posterior probabilities / bootstrap support value for Maximum likelihood. Sample names correspond to those given in Table 1. For clarity, branches that representing individuals with the same taxonomic unit were collapsed. Grey triangle refers to *M. leucogaster* and Black triangle represents to *M.cupreus*.

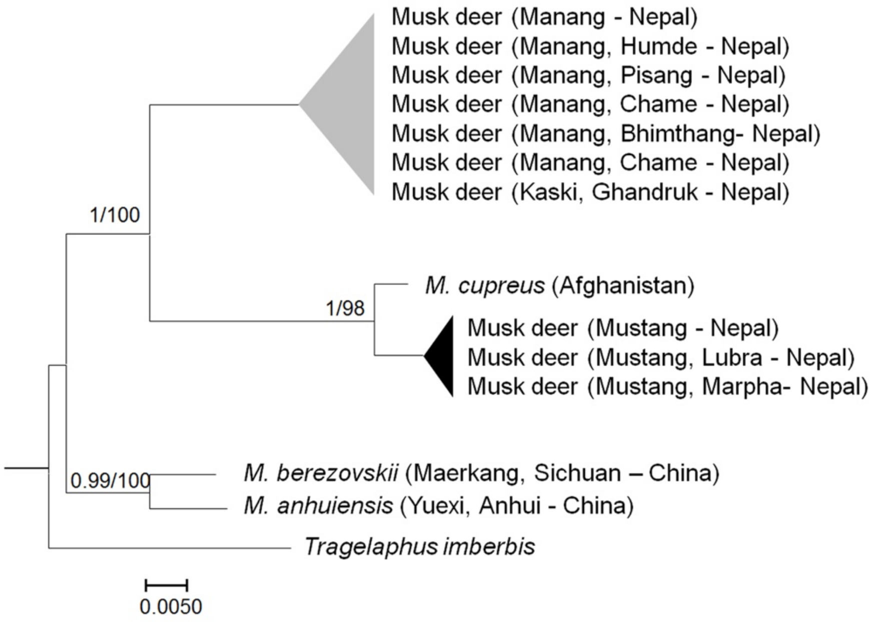

Supplementary Fig. S2. Bayesian inference (BI) tree estimated based on D-loop sequences. Values on branches of the tree show the Bayesian posterior probabilities / bootstrap support value for Maximum likelihood. Sample names correspond to those given in Table 1. For clarity, branches that representing individuals with the same taxonomic unit were collapsed. Grey triangle refers to *M. leucogaster* and Black triangle represents to *M.cupreus*.

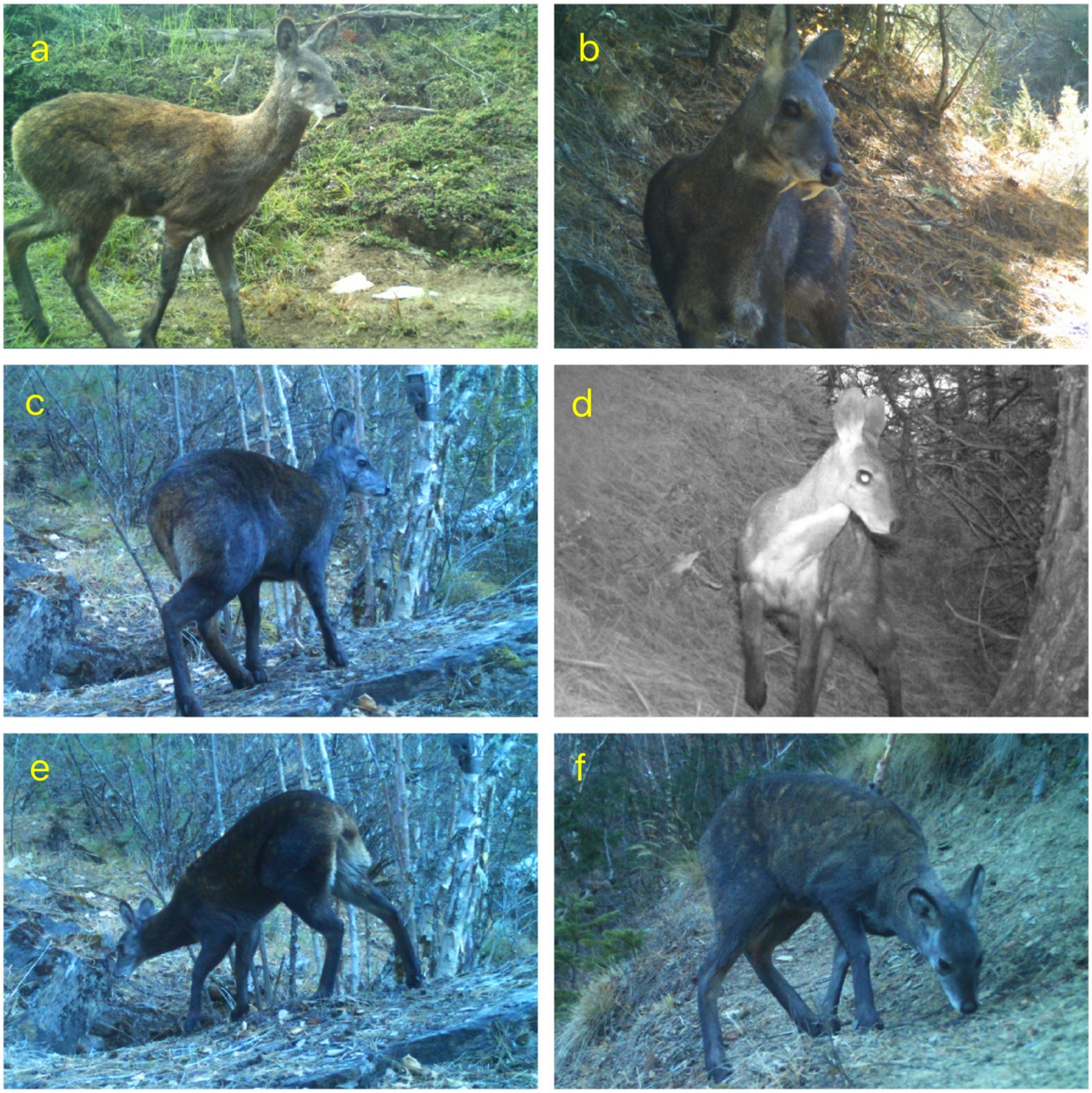

Supplementary Figs S3. Different pelage color of Himalayan musk deer photographed in Manang, Annapurna Conservation Area, Nepal during summer and winter. (a) Adult male **(b)** Adult male **(c)** Adult female **(d)** Adult male **(e)** Juvenile male **(f)** Juvenile female
